# PI3K*γ* Regulatory Protein p84 Determines Mast Cell Sensitivity to Ras Inhibition—Moving Towards Cell Specific PI3K Targeting?

**DOI:** 10.3389/fimmu.2020.585070

**Published:** 2020-10-28

**Authors:** Julie R. Jin, Elena Gogvadze, Ana R. Xavier, Thomas Bohnacker, Jan Voelzmann, Matthias P. Wymann

**Affiliations:** Department of Biomedicine, University of Basel, Basel, Switzerland

**Keywords:** inflammation, allergy, phosphoinositide-3-kinase (PI3K), PIK3CG, Ras family proteins, p101, p84, IgE (Immunoglobulin E)

## Abstract

Mast cells are the major effector cells in immunoglobulin E (IgE)-mediated allergy. The high affinity IgE receptor Fc*ε*RI, as well as G protein-coupled receptors (GPCRs) on the mast cell surface signals to phosphoinositide 3-kinase *γ* (PI3K*γ*) to initiate degranulation, cytokine release, and chemotaxis. PI3K*γ* is therefore considered as a target for treatment of allergic disorders. However, leukocyte PI3K*γ* is key to many functions in innate and adaptive immunity, and attenuation of host defense mechanisms is an expected adverse effect that complicates treatment of chronic illnesses. PI3K*γ* operates as a p110*γ*/p84 or p110*γ*/p101 complex, where p110*γ*/p84 requires Ras activation. Here we investigated if modulation of Ras-isoprenylation could target PI3K*γ* activity to attenuate PI3K*γ*-dependent mast cell responses without impairment of macrophage functions. In murine bone marrow-derived mast cells, GPCR stimulation triggers activation of N-Ras and H-Ras isoforms, which is followed by the phosphorylation of protein kinase B (PKB/Akt) relayed through PI3K*γ*. Although K-Ras is normally not activated in Ras wild-type cells, it is able to compensate for genetically deleted N- and H-Ras isoforms. Inhibition of Ras isoprenylation with farnesyltransferase inhibitor FTI-277 leads to a significant reduction of mast cell degranulation, cytokine production, and migration. Complementation experiments expressing PI3K*γ* adaptor proteins p84 or p101 demonstrated a differential sensitivity towards Ras-inhibition depending on PI3K*γ* complex composition. Mast cell responses are exclusively p84-dependent and were effectively controlled by FTI-277. Similar results were obtained when GTP-Ras was inactivated by overexpression of the GAP-domain of Neurofibromin-1 (NF-1). Unlike mast cells, macrophages express p84 and p101 but are p101-dominated and thus remain functional under treatment with FTI-277. Our work demonstrates that p101 and p84 have distinct physiological roles, and that Ras dependence of PI3K*γ* signaling differs between cell types. FTI-277 reduces GPCR-activated PI3K*γ*  responses in p84-expressing but not p101-containing bone marrow derived cells. However, prenylation inhibitors have pleiotropic effects beyond Ras and non-tolerable side-effects that disfavor further clinical validation. Statins are, however, clinically well-established drugs that have previously been proposed to block mast cell degranulation by interference with protein prenylation. We show here that Simvastatin inhibits mast cell degranulation, but that this does not occur *via* Ras-PI3K*γ* pathway alterations.

## Introduction

Mast cells are key effector cells in the pathology of allergic disease and chronic inflammation and are thus target cells of novel therapeutic strategies (1). In sensitized mice and human patients, IgE binds to the high affinity IgE receptor (FcεRI) present on the surface of tissue mast cells (2). Multivalent antigen/IgE complexes cross-link Fc*ε*RI, and trigger a protein tyrosine kinase cascade involving Src family kinase activation and Syk (spleen tyrosine kinase) translocation. The subsequent relay of signals through Bruton tyrosine kinase (Btk) to phospholipase C*γ* (PLC*γ*) culminates in an extracellular calcium influx committing mast cells to release their histamine granule contents and initiate *de novo* synthesis of pro-inflammatory and immuno-modulatory mediators, including chemokines, cytokines, growth factors, vasoactive compounds, and more (3).

We have demonstrated earlier that G-protein coupled receptor (GPCR) ligands synergize with IgE/antigen to stimulate mast cell degranulation (4–6). IgE/antigen stimulated mast cells also release adenosine, which yields an autocrine augmentation of mast cell activation *via* the G*α*_i_-coupled A3 adenosine receptor (7), triggering activation of phosphoinositide 3-kinase *γ* [PI3K*γ* (4–6)]. An alternative activation mechanism downstream of Fc*ε*RI involves the Ca^2+^-mediated activation of protein kinase C *β* (PKC*β*), which phosphorylates and activates PI3K*γ* (8).

An important aspect in anaphylaxis is recruitment of mast cell precursors to the tissue, which is also mediated by GPCRs engaging in PI3K*γ* activation (6, 9).

Mice lacking functional PI3K*γ* are thus resistant to IgE/antigen-induced anaphylaxis (4, 6), show a reduced IgE-mediated recruitment of mast cells to tissues (6), and display attenuated airway and pulmonary inflammation (10, 11), ventilator induced lung injury (12) and allergic asthma (13). PI3K*γ* therefore qualifies as a potential therapeutic target in allergic conditions.

Furthermore, PI3K*γ* is highly expressed in leukocytes of the myeloid and lymphoid lineage (14–17) and is involved in the transduction of innate and adaptive immune responses. Leukocyte chemotaxis, release of inflammatory mediators, and activation of the NADPH oxidase to release reactive oxygen species (ROS) represent crucial host defense mechanisms that require G protein-coupled receptor (GPCR) engagement and activated PI3K*γ* (4, 14–16, 18, 19). Early on, PI3K*γ* inhibition with AS-605240 has demonstrated protection against rheumatoid arthritis (20), pancreatitis (21), glomerulonephritis, and systemic lupus (22) in mouse models. Genetic and pharmacological targeting of PI3K*γ* attenuates macrophage/foam cell activation and atherosclerosis and supports plaque stability (23–25). Genetic inactivation of PI3K*γ* activity also attenuates heart failure during chronic pressure overload (26) and diet-induced obesity (27), partially reliant on kinase-independent functions of PI3K*γ* as a scaffold protein for protein kinase A and phosphorylase 3B.

The flip-side to a broad action of PI3K*γ* inhibition in various animal disease models are potential associated adverse effects, including susceptibility to infections, as indicated by reduced neutrophil (14, 19), macrophage (14, 28, 29) and dendritic cell motility (17) in PI3Kγ null cells and mice. Moreover, PI3K*γ* has been implicated in anti-viral response against Influenza A infection recently (30, 31). The possibility of cell type-specific PI3K*γ* targeting, allowing for alleviation of allergic inflammation without a general suppression of host immune defense would therefore be of great value.

PI3K*γ* acts as a heterodimer of a catalytic p110*γ* subunit and one of two possible adaptor proteins—p84 (also called p87^PIKAP^) (5, 32) or p101 (33). Both adaptor proteins take a role in the coupling of GPCR signaling to PI3K*γ*, but p101 and p84 appear to have discrete physiological functions (5, 34). Distinct pools of PtdIns(3,4,5)*P*_3_ at the plasma membrane emerging from the two PI3K*γ*/adaptor subunit complexes display a differential sensitivity to cholesterol depletion and capacity to promote mast cell granule release (5). Adaptor-specific responses were also described in neutrophils (34, 35), where p101 played a key role in cell migration, while p84 was essential for ROS production upon chemoattractant stimulation. Moreover, adaptor proteins are not equally distributed among hematopoietic cells. While lymphocytes express p101, mast cells express only the p84 adaptor subunit, but neutrophils and macrophages contain both p101 and p84 adaptors (5, 32).

Finally, a further distinction between adaptor subunits was revealed by analysis of the role of the small GTPase Ras in the activation of the PI3K*γ* complexes. Whereas p101/p110*γ* is recruited and stimulated by G*βγ* subunit of GPCRs and does not require Ras to be operational, Ras is indispensable for membrane recruitment and activation of the lipid kinase in the p84/p110*γ* complex (5, 36). Differential involvement of Ras opens new opportunities for targeted regulation of the two PI3K*γ* complexes that could provide novel ways to specifically control distinct cell responses.

In the current study, we tested whether inhibition of Ras could attenuate mast cell activation due to its involvement in p84/p110*γ* complex-dependent cell responses, and assessed if macrophages would be spared by Ras targeting.

## Materials and Methods

### Mice

Transgenic mouse strains lacking H-Ras (37), N-Ras (38) and p110*γ* (14) were previously described. Mice were backcrossed to a C57BL/6J background and housed according to the institutional guidelines. In all experiments 8–12-week-old male and female animals were utilized. All animal experiments were carried out in accordance with the guidelines of the Swiss Federal Veterinary Office (SFVO) and the Cantonal Veterinary Office of Basel-Stadt (license number 2143).

### Bone Marrow Derived Mast Cell (BMMC) and Macrophage (BMMØ) Culture

BMMC and BMMØ were cultured as previously described in (5). Briefly, bone marrow was isolated from femurs of wild-type and transgenic mice. To initiate differentiation of BMMCs, bone marrow cells were cultured in IMDM supplemented with 10% heat-inactivated fetal calf serum (HI-FCS), 2 mM L-Glutamine (Sigma, G7513), 100 U/ml Penicillin + 100 µg/ml Streptomycin (Sigma, N109), 50 μM *β*-mercaptoethanol (Sigma M6250) and 2 ng/ml murine interleukin-3 (IL-3, Peprotech 213-13). Recombinant murine SCF (5 ng/ml, Peprotech 250-03) was added only during the first passage. c-kit^+^FcεRI^+^ BMMCs were used for experiments after 4 weeks of culture. Culture medium was supplemented with IL-3 (2 ng/ml) every two days.

To generate BMMØ, bone marrow cells were cultured in bacterial petri dishes (Greiner bio-one 633180) in RPMI supplemented with 10% HI-FCS, 2 mM L-Glutamine, 1% Penicillin/Streptomycin, 50 μM *β*-mercaptoethanol and 20% L929-conditioned medium. Non-adherent cells were collected after 5 days of culture to perform experiments.

For BMMC and BMMØ GPCR activation adenosine (Fluka Bio Chemika 01890), recombinant mouse C5a (R&D Systems #8085-C3-025) and platelet activating factor (PAF; Sigma, P7568) were used.

### Ras Activation Assay and Western Blot Analysis

For pull-down of activated GTP-Ras 20 × 10^6^ cells per experiment were starved in IMDM 2% HI-FCS (BMMC) or RPMI 1% HI-FCS (BMMØ) for 3 h. After starvation, cells were resuspended in Hank’s Balanced Salt Solution (15 mM HEPES, 140 mM NaCl, 5 mM KCl, 2.8 mM NaHCO_3_, 1.5 mM CaCl_2_, 1 mM MgCl_2_, 0.06 mM MgSO_4_, pH = 7.4) supplemented with 1% BSA and 60 mM Glucose. Stimulation with 4 µM adenosine (Sigma 01890) or 10 nM murine recombinant C5a (R&D Systems 2150-C5-025) was terminated after 2 min by placing tubes on ice, spinning down, and removing supernatant. Subsequently cells were incubated in 1 ml lysis buffer (50 mM Tris pH 7.5, 150 mM NaCl, 5 mM MgCl_2_, 1 mM EGTA, 1% NP-40) supplemented with protease inhibitors (Leupeptin, Pepstatin, Aprotinin, PMSF, DTT, DFP) and containing GST-tagged Ras-binding domain of Raf1A (20 µg/reaction) and 100 µM GDP. After 10 min incubation on ice, lysates were centrifuged at 14,000 g for 15 min. For total lysis aliquots (input), 80 µl of the lysate was mixed with 20 µl 5× sample buffer (125 mM Tris-HCl (pH 6.8), 4% SDS, 10% *β*-mercaptoethanol, 20% glycerol and bromophenol blue) before denaturation (96°C, 7 min) and protein separation by SDS-PAGE, and transferred to a PDVF membrane (Immobilon FL, Millipore IPFL0010).

The remaining lysate was incubated with 40 µl 50% Glutathione-Sepharose 4B bead slurry in lysis buffer (GE Healthcare, 17-0756-01) for 2 h at 4°C. Beads were resuspended in 20 µl 2× sample buffer; denatured and pulled-down Ras protein was subjected to SDS-PAGE immunoblotting. The list of western blot antibodies is provided in the supplementary section (**Table S3**).

Recombinant p84 and p101 kindly provided by R. Williams were used as standards to quantify expression levels of PI3K*γ* adaptor proteins on western blots.

### Mast Cell Degranulation Cytokine Expression and RT-qPCR

*β*-hexosaminidase assay to assess mast cell degranulation was performed as previously described in (5). For measurement of cytokine expression, BMMCs were pre-loaded with IgE anti-DNP-HSA (clone SPE-7, Sigma D8406) for 16 h, washed, and resuspended in fresh IMDM complete supplemented with IL-3 (2 ng/ml). Subsequently, cells were incubated with DNP-HSA (2 ng/ml, Sigma D8406) and adenosine (2 µM) for 6 h at 37°C 5% CO2, and RNA was extracted using RNeasy Mini Kit (Qiagen 74104).

2 µg total RNA was used for reverse transcription with M-MVL RTase and oligo dT primers. Quantitative polymerase chain reaction (qPCR) was performed on StepOnePlus Real-Time PCR System (Applied Biosystems) with MESA Green qPCR MasterMix Plus for SYBR Assay (Eurogentech). qPCR results of Ras isoforms and farnesyltransferases (FTases) were normalized to GAPDH according to the following formulas

$$\%\;\text{of GAPDH}=2^{-\text{dCT}}\times 100\;\text{and dCT}=CT_{\text{target}}-CT_{\mathrm{GAPDH}}$$

All primer sequences for qPCR are listed in the supplementary section (**Table S1**). Relative RNA expression was normalized to GAPDH as endogenous control and WT unstimulated cells as reference sample.

$$\text{Fold change}=2^{-\text{ddCT}}\;\text{and}\;\text{ddCT}=\text{dC}T_{\mathrm{sample}}-\text{dC}T_{\text{reference}}$$

### BMMC and HEK-293 Transfection

BMMCs were transfected with Amaxa Nucleofector kit T (VCA-1002) using 14 µg of total plasmid per 10 × 10^6^ cells. Medium was changed 5 h after transfection, and cells were cultured overnight in IMDM complete supplemented with IL-3 (2 ng/ml). Plasmids encoding for p101, p84, p110*γ*, and NF1 were previously utilized in (5) and (36). Plasmid sequences of codon-optimized H-Ras, N-Ras, and K-Ras for transfection in human embryonic kidney 293 (HEK293) cells are provided in the supplementary section (**Table S2**). 24 h prior to the transfection, HEK293 cells cultured in DMEM supplemented with 10% HI-FCS, 2 mM L-glutamine (Sigma, G7513), 100 U/ml Penicillin/Streptomycin (Sigma, N109) were seeded on a 6 cm dish. The following day, 2.5 ml medium was replaced before addition of the mixture of 3 μg total DNA and 6 μl JetPEI transfection solution (Polyplus, 101B-010) in 100 μl 150 mM NaCl. 6 h post-transfection, we added fresh medium and cultured cells overnight until lysis for protein determination or imaging of Ras sub-cellular localization on a Leica DMI6000 microscope with Photometrics CoolSnap HQ2 camera.

### Transwell Migration

250,000 cells were placed into fibronectin-coated Transwell chambers (0.5 μm pores, Corning N32421) with migration assay medium (IMDM or RPMI, 2 mM L-Glutamine, 0.1% BSA low endotoxin, 1% Penicillin–Streptomycin, 50 µM *β*-mercaptoethanol, 20 mM HEPES pH 7.4) containing 1 µM adenosine (BMMC) or 10 nM C5a (BMMØ) as chemoattractant in the lower well. After 6 h at 37°C and 5% CO_2_ Transwell chambers and lower well compartments were fixed with Paraformaldehyde (PFA) 4% and stained with Hoechst nuclear stain (Invitrogen, H1399). Migrated cells were detected by fluorescence microscopy (Leica DMi8, DFC 9000 GT camera) and counted using the FIJI image analysis software. GFP fluorescence was used to detect transfected cells.

### Statistics

All data is presented as mean ± standard error of mean (SEM) of n ≥ 3 biological replicates from independent experiments. The exact number of individual experiments is stated in the figure legends. Student’s t-test or one-way ANOVA with Bonferroni’s *post hoc* test (GraphPad prism) were used for calculation of p-values as indicated for each panel (ns: p > 0.05; *: p ≤ 0.05, **: p ≤ 0.01, ***: p ≤ 0.001; ****: p ≤ 0.0001).

## Results

### N-Ras and H-Ras Are Activated Downstream of GPCRs in Mast Cells

**Figure 1:**
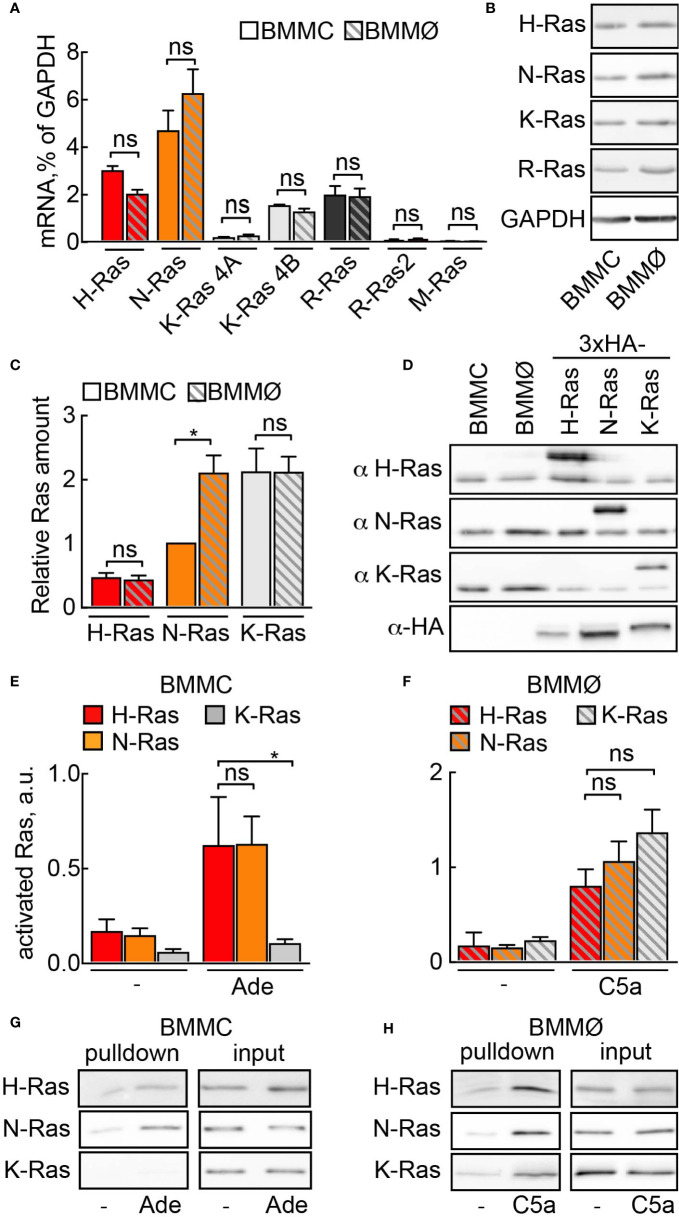
Ras activation by GPCR ligands. **(A)** Relative mRNA abundance of N-Ras, K-Ras 4A, K-Ras 4B, H-Ras, R-Ras, R-Ras2 and M-Ras was assessed in BMMCs and BMMØs by qPCR and normalized to GAPDH expression level (n = 5–6). **(B)** N-Ras, K-Ras, H-Ras and R-Ras proteins were detected in BMMC and BMMØ lysates by Western blotting using corresponding isoform-specific antibodies. **(C)** Quantification from n = 7 measurements of Ras isoforms in BMMCs and BMMØs, with N-Ras expression used as a reference point (=1). **(D)** Plasmids encoding HA-tagged codon-optimized N-, K-, and H-Ras were transfected into HEK293 cells. Protein expression levels of the three isoforms in HEK293 lysates were equalized using anti-HA antibodies. These lysates were then used as standards for quantification of relative amount of Ras isoforms in **(C)**. **(E–H)** Ras activation assay in BMMCs and BMMØs. Cells were starved in the corresponding starvation medium for 4 h before stimulation with 4 µM adenosine (Ade) (BMMC) or 10 nM C5a (BMMØ). GST-tagged Ras binding domain (RBD) of Raf1A was used to pull down GTP-loaded activated Ras. N-Ras, K-Ras, and H-Ras were subsequently detected in the pulled down fraction by Western blotting using isoform-specific antibodies and presented as a percentage of total amount of the corresponding isoform in the lysate used for the pull-down experiment (input). **(E, F)** show quantifications of n = 5**–**14 pull-down assays. Representative Western blot images are presented in **(G, H)**. Statistical analysis was performed with one-way ANOVA and Bonferroni correction. Significance levels in all figures are indicated as non-significant (ns): p > 0.05; *p ≤ 0.05, **p ≤ 0.01, ***p ≤ 0.001; ****p ≤ 0.0001.

First, we investigated which Ras isoforms would qualify for PI3K*γ* signaling in mast cells. Three Ras genes encode for four protein homologs N-Ras, H-Ras, K-Ras4A, and K-Ras4B (38). We found similar expression of N-Ras, K-Ras4B, H-Ras, and R-Ras (Ras-related protein) at the mRNA and protein level in mast cells and macrophages (**Figures 1A, B****)**. In order to estimate the relative ratio between these isoforms, we expressed codon-optimized 3×-HA tagged N-, K-, and H-Ras in HEK293 cells and used transfected HEK293 cell lysates as standards for protein quantification (**Figures 1C, D****)**. In BMMCs, K-Ras protein was the most abundant isoform, with expression levels twice as high as N-Ras and four times as high as H-Ras. In macrophages, N- and K-Ras levels were equal, while H-Ras was four times less abundant.

To determine which Ras isoform is activated downstream of GPCRs we performed GTP-Ras pull-down assays using the Ras-binding domain (RBD) of Raf. The C5a receptor [C5a anaphylatoxin chemotactic receptor 1 (C5AR1), CD88] expressed on macrophages responds to C5a stimulation and activates PI3K*γ* (14, 28). All three Ras isoforms (N-, K-, and H-Ras) were GTP-loaded after macrophage stimulation with C5a (**Figures 1F, H****)**. In mast cells, however, stimulation with adenosine activates PI3K*γ* downstream A3AR (4, 7). Adenosine led to the selective activation of N- and H-Ras, but not K-Ras (**Figures 1E, G****)**, suggesting that K-Ras is normally not involved in GPCR-mediated activation of PI3K*γ* in mast cells.

### Loss of N- and H-Ras -Compensated by Upregulation of Substitute Ras Isoforms

**Figure 2:**
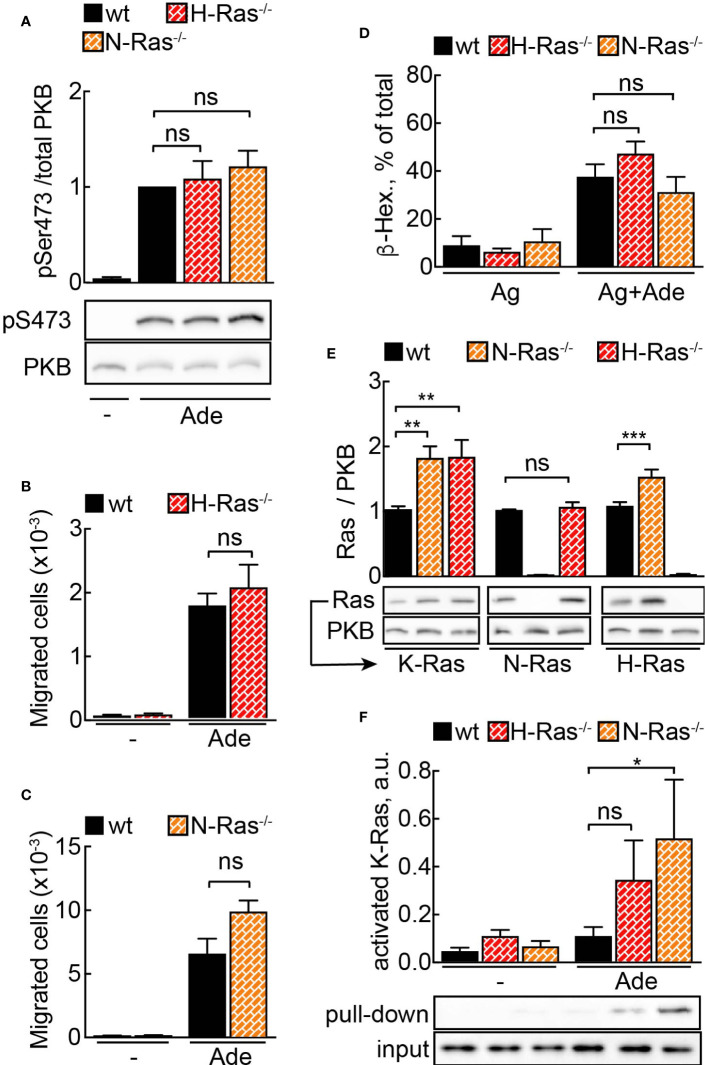
N- or H-Ras deletion without effect on PI3K*γ* signaling due to Ras isoform compensation. **(A)** WT, H-Ras^−/−^ and N-Ras^−/−^ BMMCs were starved in IL-3-free medium containing 2% FCS for 4 h and stimulated with 2 µM adenosine for 2 min at 37°C. Phosphorylation of PKB at Ser473 was determined by Western blotting and normalized to the total PKB levels (n = 8–12). **(B, C)** Migration of wild-type (WT), H-Ras^−/−^ and N-Ras^−/−^ cells was assessed in Transwell chambers for 6 h at 37°C with 2 mM Ade in the lower well (n = 5–8). **(D)** Wild-type (WT), H-Ras^−/−^, and N-Ras^−/−^ BMMCs were loaded overnight with anti-DNP IgE (100 ng/ml) followed by stimulation with DNP-HSA (antigen, Ag, 2 ng/ml) alone or in combination with 2 µM Ade. Release of *β*-Hexosaminidase was quantified 20 min after stimulation (n = 3–9). **(E)** K-Ras, N-Ras and H-Ras proteins were detected in the lysates from WT, N-Ras^−/−^ or H-Ras^−/−^ BMMCs by Western blotting using isoform-specific antibodies and normalized to the corresponding protein amount in WT cells (n = 8–15). **(F)** WT, N-Ras^−/−^ and H-Ras^−/−^ BMMCs were starved in IL-3-free medium containing 2% FCS for 4 h and stimulated with 4 µM Ade for 1 min at 37°C. Active Ras was pulled down from the lysates using GST-tagged Ras binding domain of Raf1A protein, detected by Western blotting with isoform-specific Ras antibodies and presented as a percentage of the total amount of the corresponding Ras isoform in the lysate used for the pull-down experiment (n = 3–9). Statistical analysis was performed with one-way ANOVA for **(A–E)**, and Student’s t-test for **(F)**.

To further study the physiologic importance of N-Ras and H-Ras isoforms for mast cell activity, we derived mast cells from N-Ras^−/−^ and H-Ras^−/−^ mouse bone marrow. Unexpectedly, neither adenosine-induced signaling to PKB/Akt (**Figure 2A**) nor PI3K*γ*-dependent migration (**Figures 2B, C****)** or degranulation (**Figure 2D**) was affected in any of the analyzed genotypes.

However, quantification of Ras proteins revealed an upregulation of alternative Ras isoforms in the knock-out cells (**Figure 2E**). The level of K-Ras was elevated twice in N-Ras^−/−^ and H-Ras^−/−^ cells as compared to wild-type BMMCs. H-Ras showed approximately a 1.5-fold increase in N-Ras^−/−^ cells. Interestingly, N-Ras was not upregulated in H-Ras^−/−^ BMMCs. The fact that K-Ras activation occurs only significantly in the absence of N-Ras or H-Ras without any loss of cellular responsiveness illustrates the ability of K-Ras to compensate for absence of N-Ras or H-Ras (**Figure 2F**). Altogether this demonstrates that a major part of the GPCR signal is relayed *via* N-Ras and H-Ras, but that a dynamic compensatory redundancy of Ras isoforms exists in mast cells.

As all Ras proteins have to undergo post-translational isoprenylation to enable stable lipid membrane anchoring, the revealed redundancy between N-Ras, H-Ras, and K-Ras was not expected to interfere with the action of farnesyltransferase inhibitors (FTIs) to achieve a pharmacological Ras inhibition in mast cells.

Isoprenylation inhibitors prevent the addition of farnesyl (FTIs) or geranylgeranyl residues (GGTIs) to the CaaX box motif at the C-terminus of small GTPases, thus detaching these proteins from cell membranes (39–41). FTI-277 was used here because of its excellent selectivity for FTase over GGTase I (42).

**Figure 3:**
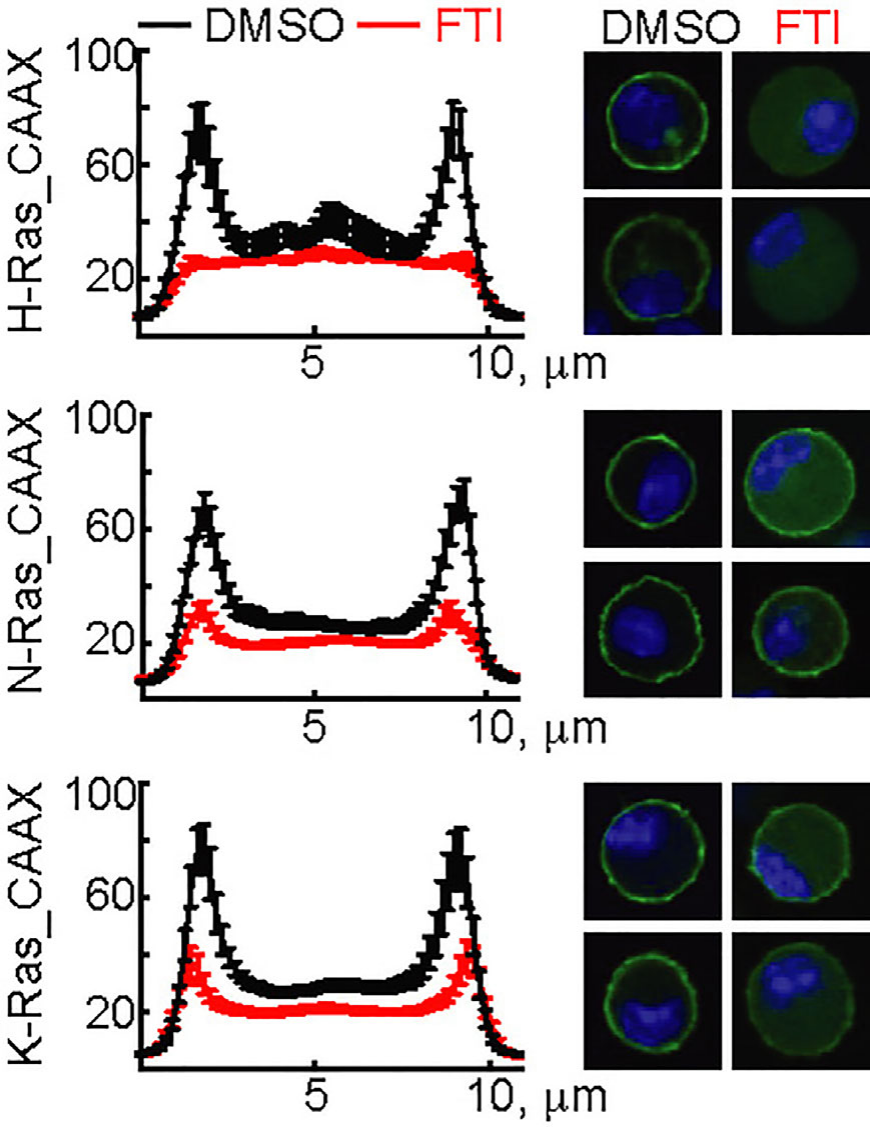
Action of FTI-277 on Ras membrane localization. BMMCs were transfected with constructs expressing GFP-tagged CAAX-domain (C-terminal 25 amino acids) of H-Ras, N-Ras or K-Ras and treated with 5µM FTI-277 for 72 h. Cells were fixed in 4% p-formaldehyde, and images were acquired by confocal microscopy. The image squares have a length of 10 µm. Left side graphs show distribution of Ras protein along a cross-section of the cell. For each condition n ≥ 18 cells were analyzed. Plotted are mean values ± standard error of mean (SEM).

The three N-, H-, and K-Ras isoforms only differ in the 25 C-terminal amino acids containing the CaaX-box motif. To validate the action of FTI-277, GFP-constructs using the N-, H-, and K-Ras CaaX-box sequences were transfected into mast cells. FTI-277 caused delocalization of all GFP-CaaX constructs from the plasma membrane to the cytosol, resulting in complete displacement of H-Ras and reduction of N- and K-Ras in the cortical regions (**Figure 3**).

**Figure 4:**
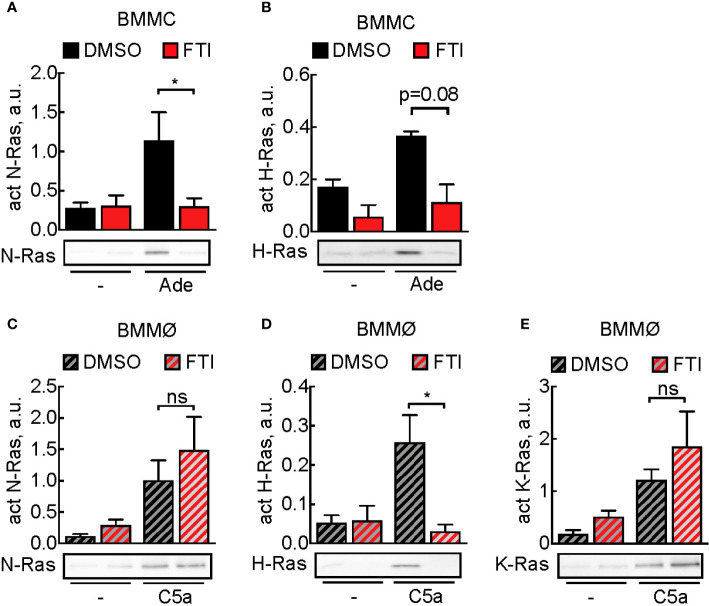
Prevention of activation of Ras isoforms by FTI-277. **(A, B)** Effect of FTI-277 on N-Ras and H-Ras activation in BMMCs. Cells were treated with DMSO or 5 µM FTI-277 for 72 h. Activated N-Ras and H-Ras was pulled down with GTP-Raf-RBD and normalized to Ras amount in complete cell lysate. **(C–E)** Effect of FTI-277 on N-Ras, K-Ras, and H-Ras activation in BMMØs. BMMØs cultured for 5 days were treated with DMSO or 5 µM FTI-277 for 72 h. Every quantification contains n = 3–6 Ras activation assays, and one representative immunoblot is shown for each condition. Student’s t-test was applied for statistical analysis.

Subsequently, we assessed the effect of FTI-277 on Ras activation: in mast cells, adenosine-induced N-Ras activation was completely blocked, and H-Ras activation was decreased, although not statistically significant (**Figures 4A, B**). In macrophages, N-, H-, and K-Ras were activated upon stimulation with C5a, but here FTI-277 only inhibited activation of H-Ras (**Figures 4C–E)**.

To determine why macrophages remained relatively insensitive towards FTI-277, expression levels of prenyltransferases before and after inhibitor exposure were determined. Treatment with FTI-277 did not cause, however, significant differences in the expression of prenyltransferases in neither macrophages nor mast cells (**Figures S2B, C**). The accumulation of prelamin A confirmed that protein farnesylation was effectively blocked under the applied incubation conditions. The fact that FTI-277 had no effect on Rap1A geranyl-geranylation, excludes that FTI-277 affected geranyl-geranylation in BMMC and BMMØ (**Figure S1**).

### Ras Inhibition With FTI-277 Leads to Reduced PI3K*γ* Signaling in Mast Cells

**Figure 5:**
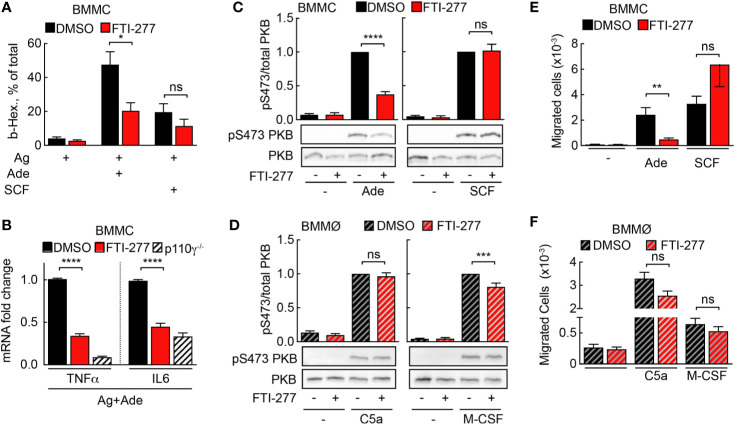
Mast cell but not macrophage activation is affected by FTI-277-mediated Ras inhibition. **(A)** DMSO- or FTI-277-treated WT-BMMCs were loaded overnight with anti-DNP IgE (100 ng/ml) followed by stimulation with DNP-HSA (Ag, 2 ng/ml) alone or in combination with 2µM Ade or 10 ng/ml SCF. Release of *β*-Hexosaminidase was quantified 20 min after stimulation (n = 6–7). **(B)** DMSO- or FTI-277-treated WT-BMMCs as well as p110*γ*^−/−^ BMMCs were exposed overnight to anti-DNP IgE (100 ng/ml) followed by stimulation with DNP-HSA (Ag, 2 ng/ml) together with 1 µM Ade for 6 h. TNF-α and IL-6 expressions were determined by qPCR and normalized to the level of GAPDH expression. Fold change of expression in FTI-277 treated or p110*γ*^−/−^ cells was quantified relative to DMSO-treated control (n = 6). **(C, D)** BMMCs and BMMØs were treated with either DMSO or 5 µM FTI-277 for 72 h, starved for 4 h, and activated with 2 µM Ade, 10 ng/ml SCF, 10 nM C5a, or 30 ng/ml M-CSF for 2 min at 37°C. Phosphorylation of PKB at Ser473 was determined by Western blot analysis of cell lysates with anti-PKB-Ser473 antibodies and normalized to the total level of PKB (n = 9–18). **(E, F)** Migration of BMMCs and BMMØs was assessed in Transwell chambers for 6 h at 37°C with indicated stimuli in the lower well, followed by quantification of migrated cells (n = 6–16). **(E, F)** were assessed with Student’s t-test and **(B–D)** were subjected to one-way ANOVA with Bonferroni’s *post hoc* test.

Mast cells are the main effector cells during acute IgE-dependent allergic reactions such as anaphylaxis. Activation of IgE-sensitized mast cells with allergen leads to release of inflammatory mediators from pre-formed granules. Simultaneously, *de-novo* synthesis of cytokines, chemokines, and other compounds is initiated. Importantly, secretion of TNF-α supports adhesion of rolling blood leukocytes to endothelia by upregulation of the adhesion molecule VCAM-I (6). FTI-277 attenuated degranulation of IgE/antigen-activated mast cells upon co-stimulation with adenosine, but not with stem cell factor (SCF), which signals through c-kit and PI3K*δ* (**Figure 5A**). Furthermore, p110*γ*-dependent expression of TNF-α and IL-6 was significantly decreased in mast cells co-stimulated with IgE/antigen and adenosine (**Figure 5B**).

Next, BMMCs and BMMØs were treated with FTI-277 (5 μM for 72 h) to assess phosphorylation of the main PI3K downstream target PKB/Akt. To distinguish between Ras- and Ras-independent PKB activation, BMMCs and BMMØs were stimulated with GPCR ligands after exposure to FTI-277: adenosine stimulation leads to PI3K*γ* activation *via* adenosine receptor A3 (A3AR, ARA3) in mast cells (4, 7), and C5a stimulates PI3K*γ* signaling *via* C5aR (CD88) in macrophages (14). Receptor tyrosine kinase (RTK) ligands such as SCF and macrophage colony-stimulating factor (M-CSF) served as reference for PI3K*γ*-independent signaling to PKB/Akt. In mast cells, FTI-277 led to a significant decrease in PKB/Akt phosphorylation at Ser473 upon activation with adenosine but had no effect on PKB/Akt activation upon stimulation with SCF. This demonstrates that the inhibitory action of FTI-277 is specific for the GPCR-mediated activation of PI3K*γ* (**Figure 5C**) but does not affect signaling through PI3Kδ to PKB/Akt (43). In contrast to mast cells, Ras inhibition did not affect C5a-induced phosphorylation of PKB in macrophages (**Figure 5D**).

The IC_50_ for inhibition of PKB phosphorylation by FTI-277 in adenosine-stimulated BMMCs was 1.65 µM for pSer473 and 1.61 µM for pThr308, and 4.76 µM for phosphorylation of mitogen-activated protein kinase (MAPK, also known as ERK1&2; **Figure S4**). PKB phosphorylation downstream of PI3K*γ* is therefore more sensitive towards FTI-277 as compared to MAPK, which might be attributed to a proposed multi-step cascade activation of MAPK (44).

Chemoattractant-mediated leukocyte recruitment to inflamed tissues is initiated by GPCR engagement and PI3K*γ* activation. Here, we assessed the effect of Ras inhibition on mast cell and macrophage migration *in vitro* in a Transwell migration assay. In line with the results for PKB phosphorylation, only adenosine-stimulated migration of mast cells was significantly impaired by FTI-277 (**Figure 5E**). Meanwhile, macrophage migration toward GPCR agonist C5a, as well as the RTK ligand M-CSF remained intact (**Figure 5F**).

### GGTI-298 Inhibits Mast Cell Activation

**Figure 6:**
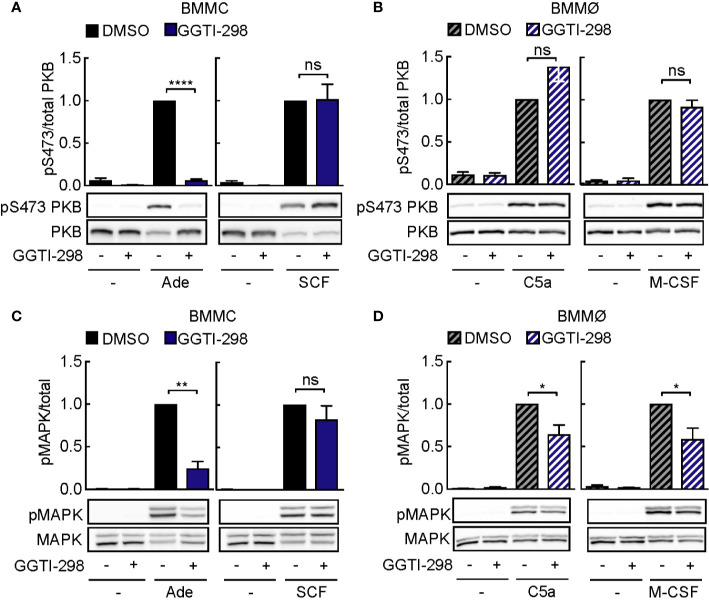
GGTI-298 affects activation of mast cells, but not macrophages. BMMCs **(A, C)** and BMMØs **(B, D)** were treated with either DMSO or 5 µM GGTI-298 for 24 h, starved for 4 h and activated with 2 µM Ade, 10 ng/ml SCF, 10 nM C5a or 30 ng/ml M-CSF for 2 min at 37°C. Phosphorylation of PKB (Ser473) and MAPK was determined by immunoblot blot analysis of cell lysates and normalized to the total level of corresponding proteins (n = 4–10). Statistical significances were assessed by Student’s t-test.

Other post-translational modifications such as geranyl-geranylation are known to provide lipid anchoring in membranes. Inhibition of geranyl-geranylation with GGTI-298 also interferes with PI3K*γ* signaling: interestingly, GGTI-298 blocked PKB phosphorylation in mast cells, but not macrophages (**Figures 6A, B****)**. Still, phosphorylation of mitogen-activated protein kinase (MAPK) was inhibited in adenosine-stimulated mast cells (**Figure 6C**) and macrophages exposed to C5a or M-CSF (**Figure 6D**). Although Ras proteins have previously not been reported to be geranyl-geranylated under normal conditions, we observed that GGTI-298 treatment causes re-localization of GFP-tagged H-Ras (**Figure S3**). N-Ras and K-Ras did not translocate to the cytosol under the same conditions. The specific action of GGTI-298 on geranyl-geranylation was confirmed by the accumulation of un-geranyl-geranylated Rap1A, while prelamin A did not accumulate in its non-farnesylated form in mast cells and macrophages (**Figure S1**).

Altogether, these results suggest that GGTI-298 does not affect PI3K*γ* signaling in mast cells by interference with Ras activation, but is likely to intercept upstream activation by geranyl-geranylated G*γ* subunit subtypes, which seems to be set up differently in macrophages.

### Statins Inhibit Mast Cell Degranulation Independently of PI3K*γ*

Statins inhibit HMG-CoA reductase, the rate limiting enzyme of cholesterol biosynthesis, and deplete farnesyl pyrophosphate (FPP). The lack of substrate for FTase and GGTase then reduces protein farnesylation and geranyl-geranylation. It has been previously reported that statins inhibit mast cell cytokine production (45) and degranulation (46), but no mechanistic explanations are available. Recent studies have demonstrated that statins also reduce disease activity of rheumatoid arthritis (47–49), illustrating immunomodulatory effects of statins.

**Figure 7:**
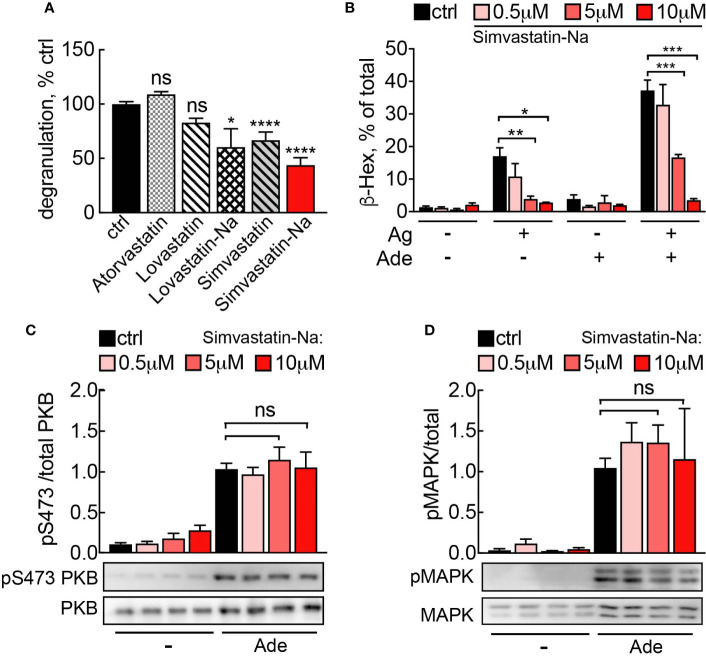
**(A)** Simvastatin inhibits mast cell degranulation without interfering with PI3K*γ* signaling. BMMCs were treated with indicated statins at 5 µM for 16 h and then loaded with anti-DNP IgE (100 ng/ml). The following day, cells were stimulated with DNP-HSA (Ag, 2 ng/ml) plus 2 µM adenosine. Release of *β*-Hexosaminidase was quantified 20 min after stimulation and normalized to untreated control (ctrl= DMSO 0.05% for Lovastatin and Simvastatin; PBS 0.001% MetOH for Atorvastatin). Lovastatin (n = 4–7) and Simvastatin (n = 10–16) are pro-drugs, while Atorvastatin (n = 4), Simvastatin-Na (n = 10–16) and Lovastatin-Na (n = 4–7) are active drugs. **(B)** Dose dependency for Simvastatin-Na was tested in three independent experiments with total n = 9 biological replicates. Degranulation was assessed with IgE/antigen-stimulation alone (Ag, DNP-HSA 2 ng/ml) and IgE/antigen co-stimulation with adenosine (2 µM). **(C, D)** Western blot analysis of BMMCs treated with 5µM Simvastatin-Na for 16 h and starved for 4 h. Adenosine (2 µM) was used as stimulus. Phosphorylated PKB and MAPK was quantified from n = 3–9 experiments. Statistical significance was tested with one-way ANOVA applying Bonferroni correction.

We therefore investigated, whether statins could interference with the Ras-PI3K*γ* signal pathway and thereby elicit anti-inflammatory actions. Among the statins tested, Simvastatin and its active derivate Simvastatin Sodium Salt (Simvastatin-Na) had the most pronounced effect on mast cell degranulation (**Figure 7A**). Simvastatin-Na decreased IgE-antigen-mediated degranulation and IgE-antigen/adenosine co-stimulation in a concentration dependent fashion (**Figure 7B**). However, decreased degranulation did not correlate with changes in Ras or PI3K*γ* pathway activation since PKB and MAPK phosphorylation remained unaffected even at elevated concentrations of Simvastatin-Na (**Figures 7C, D****)**, suggesting that Simvastatin blocks degranulation by pleiotropic action and not specific interference with PI3K*γ*.

### Sensitivity to Ras Inhibition Is Largely Determined by PI3K*γ* Adaptor Subunit

**Figure 8:**
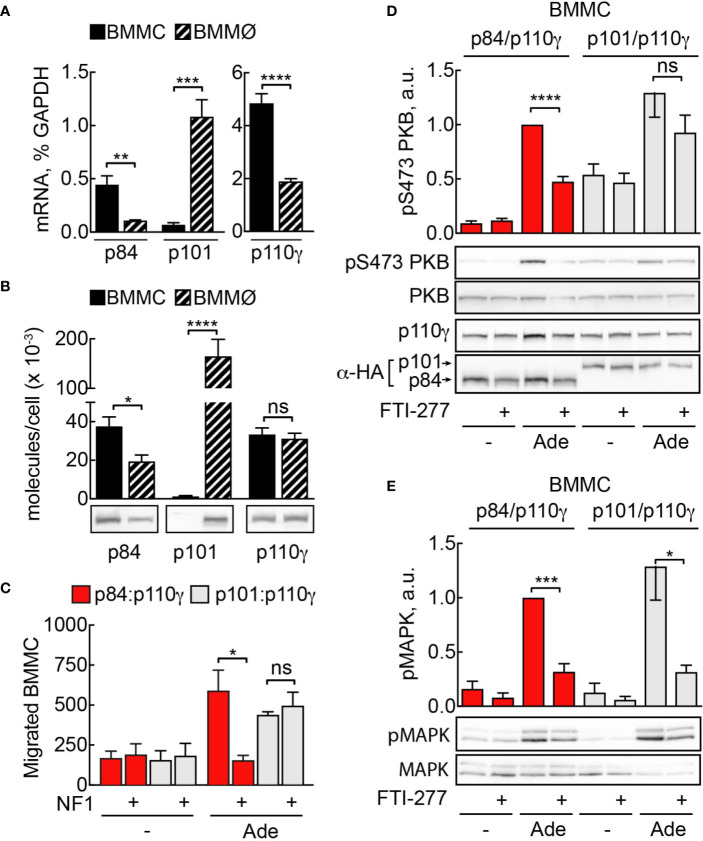
Sensitivity to Ras inhibition is defined by PI3K*γ* adaptor subunit. **(A)** Expression of p110*γ*, p84, and p101 in BMMCs and BMMØs was assessed by qPCR and normalized to GAPDH expression in BMMCs. **(B)** p84, p101, and p110*γ* proteins were detected in BMMC and BMMØ lysates by Western blotting and quantified using recombinant p84/p110*γ* and p101/p110*γ* complexes with known protein concentration. **(C)** p110*γ* and HA-p84 or HA-p101 were co-expressed with or without Flag-NF1 (GAP domain) in p110*γ*^−/−^ BMMCs; additionally, GFP-expressing plasmid was used to select for transfected cells. Migration of GFP-positive BMMCs was assayed in Transwell chambers for 6 h in the presence of 2 µM Ade in the lower well. Subsequently, GFP-positive cells were quantified by fluorescence microscopy. The panel contains n = 5 biological replicates from two independent experiments. **(D, E)** p110*γ*^−/−^ BMMCs were transfected with plasmids encoding functional p110*γ* and either HA-tagged p84 or p101. 5 h after transfection cells were put in fresh medium containing DMSO or 5 µM FTI-277. The next day, cells were starved in IL-3-free medium containing 2% FCS and stimulated with 2 µM Ade for 2 min at 37°C. Phosphorylation of PKB at Ser473 and MAPK was determined by Western blotting and normalized to the total PKB or MAPK levels, correspondingly. Transient expression of p110*γ* was assessed with anti-p110*γ* antibodies, while p84 and p101 were detected with anti-HA antibodies (n = 5–6). Student’s t-test was performed to test for statistical relationships.

PI3K*γ* adaptor subunits p84 and p101 are differentially expressed in various cell types of the myeloid and lymphoid lineages (5, 6, 32, 50). Mast cells express exclusively the p84 adaptor protein, while macrophages harbor both subunits. We measured messenger RNA by qPCR to compare expression of p101, p84, and p110*γ* between BMMC and BMMØ (**Figure 8A**). According to qPCR data, while mast cells express only p84 subunit, p101 is the dominant adaptor protein in macrophages, with its expression level exceeding p84 by tenfold (**Figure 8A**). Next, we used recombinant p84/p110*γ* and p101/p110*γ* complexes to calibrate the quantification of the corresponding proteins (**Figure 8B**). BMMCs and BMMØs possess similar amounts of p110*γ* (≈33,000 *vs* 35,000 molecules/cell). In BMMCs, the relation of p84 and p110*γ* (≈32,000 and 33,000 molecules/cell) is nearly one to one, while p101 was undetectable. Meanwhile, in BMMØ the total of p101 molecules is seven times higher compared to p84 (≈150,000 *vs* 20,000 molecules/cell).

It was previously reported that Ras is indispensable for membrane recruitment and activation of p84/p110*γ*, but not for p101/p110*γ* complexes (36). To assess if the observed difference in sensitivity to FTI-277 between mast cells and macrophages could be explained by the difference in the adaptor subunit content of these cells, we used a mast cell complementation technique described in (5): p110*γ* null BMMCs lack both p110*γ* and functional p84 and are ideal to complement either p84/p110*γ* or p101/p110*γ* complexes by nucleofection and to assess differences in signaling outputs of the two complexes in the same cellular context. We reconstituted p110*γ*^−/−^ BMMCs with p84/p110*γ* or p101/p110*γ* complexes and treated them with FTI-277 (**Figure 8D**). Only cells that expressed p84 as adaptor subunit exhibited significant reduction of phosphorylated PKB at Ser473. On the other hand, PI3K*γ* activation in cells expressing p101 adaptor protein remained insensitive to Ras inhibition. Remarkably, regardless of the adaptor protein, FTI-277 caused a significant decrease in the level of phosphorylated MAPK (**Figure 8E**). As a complementary approach for Ras inhibition, we overexpressed the GTPase-activating protein (GAP) domain of neurofibromin 1 (NF1) together with p84/p110*γ* or p101/p110*γ* in p110*γ*^−/−^ BMMCs. The ability of cells to migrate towards chemoattractant was then tested in a Transwell migration assay (**Figure 8C****)**. Like the effect of FTI-277 on PKB phosphorylation, only p84/p110*γ* containing cells lost their migratory potential after NF1 overexpression, while migration of cells expressing p101 was insensitive to Ras inactivation.

Since FTI-277 potentially affects all farnesylated proteins and does not specifically target Ras, we further excluded the possibility that the observed inhibitory action of FTI-277 on PI3K*γ* signaling is a result of impaired G-protein processing and functioning. Farnesylation of G*γ* and palmitoylation of G*α* subunits of heterotrimeric G-proteins are vital for proper GPCR signal transduction (51). In mast cells, only G*α*i-coupled GPCRs, such as adenosine A3 receptor (A3AR), have been reported to activate PI3K*γ* (4). G*α*q-coupled platelet-activating factor (PAF-) receptor triggers, however, calcium release from internal stores and phosphorylation of cyclic AMP-responsive element-binding protein (CREB) independent from PI3K*γ* (1). Treatment with FTI-277 had no effect on PAF-stimulated CREB phosphorylation in mast cells and macrophages (**Figure S5**), showing that trimeric G-protein activity remains intact upstream of PI3K*γ*.

## Discussion

### RAS Isoforms Involved in Mast Cell Activation

The importance of Ras signaling in IgE-dependent mast cell activation has been demonstrated earlier, but the nature of downstream events has remained obscured: it has been observed that the deletion of RabGEF1 leads to the overactivation of Ras and thus causes mast cell hyperresponsiveness towards Fc*ε*RI stimulation and results in severe skin inflammation with an accumulation of tissue mast cells in mice (52). Constitutive Ras activation due to deficiency of neurofibromin 1 (NF1) on the other hand, leads to hyperproliferation of mast cell-rich neurofibromas, in a situation where RTK signaling seems to dominate Ras activation (53). The importance of GPCR-induced Ras activation in mast cells however, has barely been explored, despite the physiological implications for mast cell chemotaxis and synergism with Fc*ε*RI activation during degranulation (9). PI3K*γ* in mast cells is a downstream effector of Ras and GPCRs. In the present study we exploited Ras-PI3K*γ* interactions as a proof-of-concept strategy for a cell-specific regulation of PI3K*γ* activity.

Among the seven Ras isoforms that were previously shown to interact and activate PI3Ks (N-, H-, K4A-, K4B-, R-, R1, and M-Ras (54), only N-Ras and H-Ras were found to be activated downstream of GPCRs in mast cells. N- and H-Ras, but not K-Ras, were previously reported to be associated with cholesterol-rich lipid raft domains at the plasma membrane (55). Their activation downstream of the GPCR is, therefore, in line with the lipid-raft associated activation of p84/p110*γ* complex (5). H-Ras has been proposed to be mainly localized to lipid rafts in inactive GDP-bound state and to be redistributed to non-raft microdomains of plasma membrane upon its activation (56, 57), making N-Ras the most probable candidate for p84/p110*γ* activation at the plasma membrane. However, recent studies using artificial lipid bilayer models show that in the absence of scaffolding proteins also N-Ras relocates from rafts to disordered lipid domains when switching to a GTP-bound, active state (58). But naturally, the plasma membrane of intact cells provides a very different environment that potentially contains interacting scaffolds. One possibility is that p110*γ* itself directs Ras to dedicated membrane microdomains. One could speculate that p110*γ*/p84 complex formation induces a conformational change that favors Ras binding. Analogously, allosteric effects in p110*γ* upon p101 binding have previously been proposed to increase G*βγ* affinity (59), thus minimizing the need for Ras to achieve membrane recruitment of PI3K*γ*.

Nonetheless, altering p110*γ* affinity towards Ras does not fully explain the isoform preference we observed in mast cells. Despite not being activated in wild-type mast cells, K-Ras has been activated and recruited in N-Ras^−/−^ and H-Ras^−/−^ BMMCs to restore PI3K*γ* signaling. Consequently, N-Ras^−/−^ and H-Ras^−/−^ BMMCs showed no impairment in PI3K*γ* dependent degranulation, migration, and phosphorylation of PKB. This observation clearly argues for overlapping functions of Ras isoforms present in mast cells. It remains uncertain how K-Ras is engaged only in the absence of H-Ras and N-Ras in mast cells, while in WT macrophages N-Ras, H-Ras, and K-Ras are all activated downstream of C5a stimulation.

### Pharmacologic Inhibition of Ras With FTI, GGTI, and Statins

Pharmacologic Ras inhibition with FTI-277 diminished N-Ras activation in mast cells but did not impact N-Ras and K-Ras activation in macrophages. In the case of H-Ras, we observed reduced activation in both cell types (BMMC p = 0.0863, BMMØ p = 0.0241). Alternative geranyl-geranylation of N-Ras and K-Ras in the presence of FTI-277 could have provided a hypothetical explanation for insensitivity of macrophages towards the inhibitor. According to our qPCR data, macrophages express less FTase, as well as GGTase compared to mast cells, and these expression levels were not influenced by FTI-277. This does not support the assumption that macrophages need higher dosage of FTI treatment to cope with higher amounts of FTase, neither that geranyl-geranylation is more efficient in macrophages than in mast cells. Another hypothesis is that varying half-life of Ras proteins in different cells might explain the differential susceptibility to farnesyltransferase inhibitors.

FTI-277 blocks protein farnesylation in macrophages as well as in mast cells, as controlled by accumulation of prelamin A. It is expected that farnesylated proteins other than Ras are also malfunctioning under FTI-277 treatment. Three subtypes of G*γ* subunits, G*γ*1, G*γ*8, and G*γ*11 of trimeric G-proteins are farnesylated (51). Phosphorylation of CREB in cells stimulated with platelet activating factor (PAF) however, was not attenuated (**Figure S4**). This demonstrates that G*α*q containing trimeric G-protein functions are intact. It is therefore unlikely that insufficient processing of G*γ* subunits in FTI-277 treated cells cause defective GPCR to PI3K*γ* signaling. Altogether, it appears that Ras functions are conserved more strictly in macrophages, ensuring proper host defense. Further mechanistic studies are needed to elucidate the mechanism behind the resistance of Ras proteins to FTIs in macrophages, and whether it could be used for developing strategies for cell-specific Ras targeting. Yet, a recent study by Bratt et al. (60) found that FTI-277 unexpectedly worsened asthmatic airway changes in mice instead of ameliorating them. The authors examined Ras localization in bronchial epithelial cells but did not see translocation from membrane-bound to cytosolic state. Instead, accumulation of farnesyl pyrophosphate (FPP) under FTI treatment turned out to exacerbate allergic asthma. Thus, *in vivo* FTI-277 does not act as cell-type specific agent and more importantly, did not show clinical benefits against allergic asthma.

Although Ras is not known to be geranyl-geranylated in the absence of FTIs, we observed reduced H-Ras membrane localization upon treatment with GGTI-298. We also found that inhibition of geranyl-geranylation with GGTI-298 impacted PI3K*γ* signaling. This indicates that non-Ras proteins likely affirm different sensitivity of mast cells and macrophages towards GGTIs (and FTIs) as well.

Rab proteins are geranyl-geranylated small GTPases. Rab5 in particular delivers H-Ras from the cell membrane to recycling endosomes (61, 62). Still, the di-cystein motif in Rab5’s C-terminus is modified by GGTase II (also known as Rab geranyl-geranyl transferase, RGGT) and is not expected to malfunction under treatment with the GGTase I-specific GGTI-298 (63). Several G*γ* subunit subtypes (G*γ*2–5, 7, 9, 10, 12 and 13) are geranyl-geranylated by GGTIase I and therefore likely fail to operate as relay from GPCR to PKB after GGTI-298 exposure. Despite the finding that macrophages are more resistant to GGTI-298 than mast cells, inhibition of the majority of G*γ* proteins is not a viable therapeutic approach for cell-selective PI3K*γ* inactivation.

Overall, clinical translation of prenylation inhibitors to mast cell targeted therapy is currently complicated by insufficient potency and side-effects of FTIs and GGTIs in clinical trials (64). However, inhibition of protein prenylation has previously also been postulated for statins, a class of clinically tolerable inhibitors that deplete FPP and thus lower protein prenylation. In an effort to translate our findings into therapeutic application we treated mast cells with Simvastatin, Lovastatin and Atorvastatin. Despite effective inhibition of mast cell degranulation by Simvastatin, PI3K*γ* signaling to PKB/Akt was preserved. Hence, the mast cell stabilizing effect of statins is of different nature than FTI-277 and GGTI-298 effects.

### Mast Cell Specific PI3K*γ* Targeting *via* p84 and Ras

From the discovery of the second possible adaptor subunit for PI3K*γ* (32), the questions regarding the physiological importance of having two regulatory proteins for PI3K*γ* were arising. In the recent years it has become evident that p84 and p101 are non-redundant and confer specific properties to PI3K*γ* that result in diverse cellular responses. Different outputs triggered by two complexes are most likely explained by differences in the spatiotemporal distribution of PtdIns(3,4,5)P_3_ derived from either p101/p110*γ* or p84/p110*γ* (5). Ras was shown to be indispensable for membrane recruitment and activation of p84/p110*γ*, but not p101/p110*γ* complexes (36). Ras, therefore, could contribute to the differential coupling of PI3K*γ* heterodimers to downstream responses by ensuring their distribution to dedicated membrane compartments.

The two regulatory proteins p84 and p101 are not equally distributed in PI3K*γ* expressing cells. The fact that p110*γ*^−/−^ mast cells reconstituted with p101/p110*γ* were resistant to Ras inhibition, while p84/p110*γ* mediated PI3K*γ* pathway was inhibited pharmacologically as well as by overexpression of GAP-domain of NF1, shows that PI3K*γ* adaptor subunit is a major factor explaining differential sensibility towards FTIs of p101 and p84 dominated cells. Consequently, macrophages/monocytes with an abundance of p101 adaptor protein (≈150,000 molecules/cell, ≈90% of all adaptor protein) are spared by FTIs. On the other hand, in cells with a predominant expression of p84 adaptor subunit such as mast cells, PI3K*γ*-dependent responses are susceptible to modulation of Ras signaling.

It was suggested previously that free monomeric p101 is unstable and undergoes cytosolic degradation (65). Therefore, we were surprised to detect sixfold higher abundance of p101 compared to p110*γ* in macrophages. Excess of p101 exogenously expressed was previously shown to localize to the nucleus (66). A surplus of p101 over p110*γ* and p84 in macrophages might favor p101/p110*γ* complex formation even in presence of p84.

Overall, the dominance of p84/p110*γ* complex clearly renders mast cells Ras dependent. But the currently available pharmacological inhibitors, such as FTI-277 or statins, act pleiotropically and cannot be pursued as cell-specific targeting strategy. Cell type specific modulation of PI3K*γ* might be achieved by interference with p110*γ*-adapter protein complex formation in the future. The development of specific p84/p110γ targeting strategies for mast cell-related diseases will presumably have limited effects on macrophages and other p101-dominated leukocytes, thus better preserving a proper host defense as compared to PI3K*γ* ATP-site inhibitors.

## Data Availability Statement

All datasets presented in this study are included in the article/**Supplementary Material**.

## Ethics Statement

The animal study was reviewed and approved by Swiss Federal Veterinary Office (SFVO) and the Cantonal Veterinary Office of Basel-Stadt (license number 2143).

## Author Contributions

JJ, EG, AX, TB, and JV performed experiments. JJ, EG, AX, TB, and MW analyzed data, wrote the manuscript and contributed conceptually. All authors contributed to the article and approved the submitted version.

## Funding

This work was funded by the Swiss National Science Foundation (SNF) grants 310030_153211 and 310030_189065. JJ received a Swiss Cancer Research Foundation grant (MD-PhD-3916-06-2016). EG was supported by a Marie Curie Fellowship 255070 PI3KACT and the Peter & Traudl Engelhorn Foundation.

## Conflict of Interest

The authors declare that the research was conducted in the absence of any commercial or financial relationships that could be construed as a potential conflict of interest.
