# Supplementary material for: PI3Kγ Regulatory Protein p84 Determines Mast Cell Sensitivity to Ras Inhibition—Moving Towards Cell Specific PI3K Targeting?
Source: Front Immunol. 2020 Oct 28;11:585070. doi: 10.3389/fimmu.2020.585070 (PMC7655736; doi:10.3389/fimmu.2020.585070)
Supplement: Supplementary file 1 [file DataSheet_1.pdf]

Supplementary Information

Content page

Figure S1. FTI-277 specifically inhibits protein farnesylation and GGTI-298 specifically inhibits protein geranyl-geranylation. 2

Figure S2. Expression of farnesyltransferase (FTase) and geranylgeranyltransferase (GGTase) in mast cells and macrophages. 2

Figure S3. GGTI-298 causes re-localization of H-Ras from the plasma membrane to the cytosol. 3

Figure S4. Time- and concentration-dependence of FTI-277 action on mast cells. 3-4

Figure S5. Neither FTI-277 nor GGTI-298 affect PAF-signaling in BMMC or BMMØ 4

Table S1. Primer Sequences used for qPCR 5-6

Table S2. Sequences of codon-optimized 3xHA-Ras, used for expression in HEK293 cells 7

Table S3. ORF sequences of GFP-tagged Ras isoforms used for cellular localization assays 8

Table S4. Antibodies used for immunoblotting 9-10

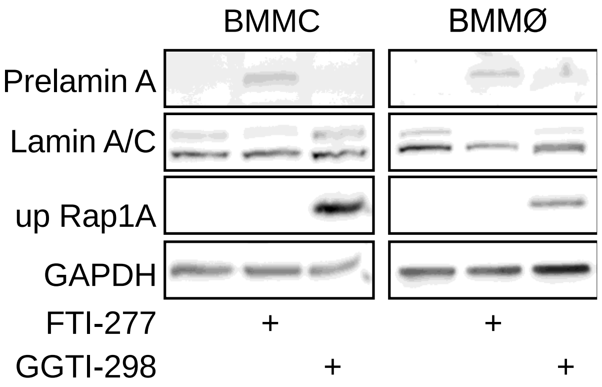

Figure S1. FTI-277 specifically inhibits protein farnesylation and GGTI-298 specifically inhibits protein geranyl-geranylation. BMMCs and BMMØ were treated with 5 µM FTI-277 (72 h) or 5 µM GGTI-298 (24 h). Levels of non-prenylated lamin (Prelamin A) and un-prenylated Rap1A (up Rap1A) were determined by Western blotting. Accumulation of Pre-lamin in FTI-277 incubated cells demonstrate inhibition of protein farnesylation. GGTI-298 had no effect on pre-lamin but abrogated geranyl-geranylation of Rap1A. Experiment was performed 3 times for each cell type and one representative Western blot image is shown.

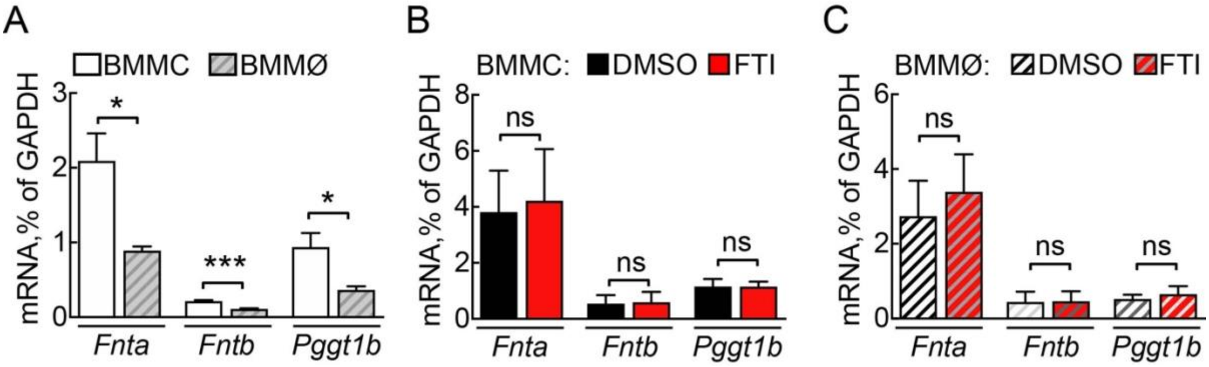

Figure S2. Expression of farnesyltransferase (FTase) and geranylgeranyltransferase (GGTase) in mast cells and macrophages. (A) mRNA abundance of *Fnta* (α subunit of FTAse and GGTase), *Fntb* (β subunit of FTAse) and *Pggt1b* (β subunit of GGTase) in BMMCs and BMMØ was assessed by qPCR and normalized to the corresponding level of GAPDH expression. Results from n=4 independent experiments are shown. **(B and C)** Effect of FTI-277 on FTase and GGTase expression. BMMCs (B) and BMMØ (C) were treated with DMSO or 5µM FTI-277 for 72 h. Expression of *Fnta*, *Fntb* and *Pggt1b* genes was assessed by qPCR and normalized to GAPDH. Experimental number is n=3 for BMMC and n=2 for BMMØ from independent experiments. Statistical significance was evaluated with Student T-test. ns: $p>0.05$; *: $p\leq0.01$, ***: $p\leq0.001$.

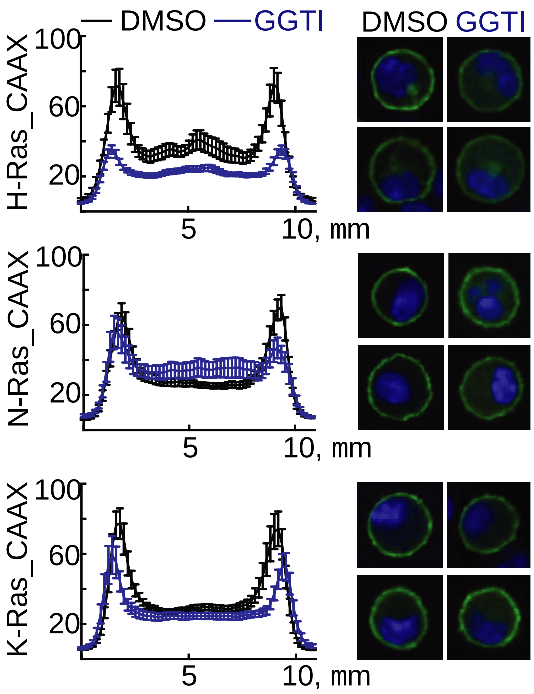

Figure S3. GGTI-298 causes re-localization of H-Ras from the plasma membrane to the cytosol. BMMCs were transfected with GFP-tagged C-terminal 25 amino acids (CAAX domain) of H-Ras, N-Ras or K-Ras. Afterwards we treated them with 5µM GGTI-298 for 24 h. Images of PFA-fixed cells were acquired by confocal microscopy. Plotted are GFP-intensity mean values ± standard error of mean (SEM) of N>18 cells.

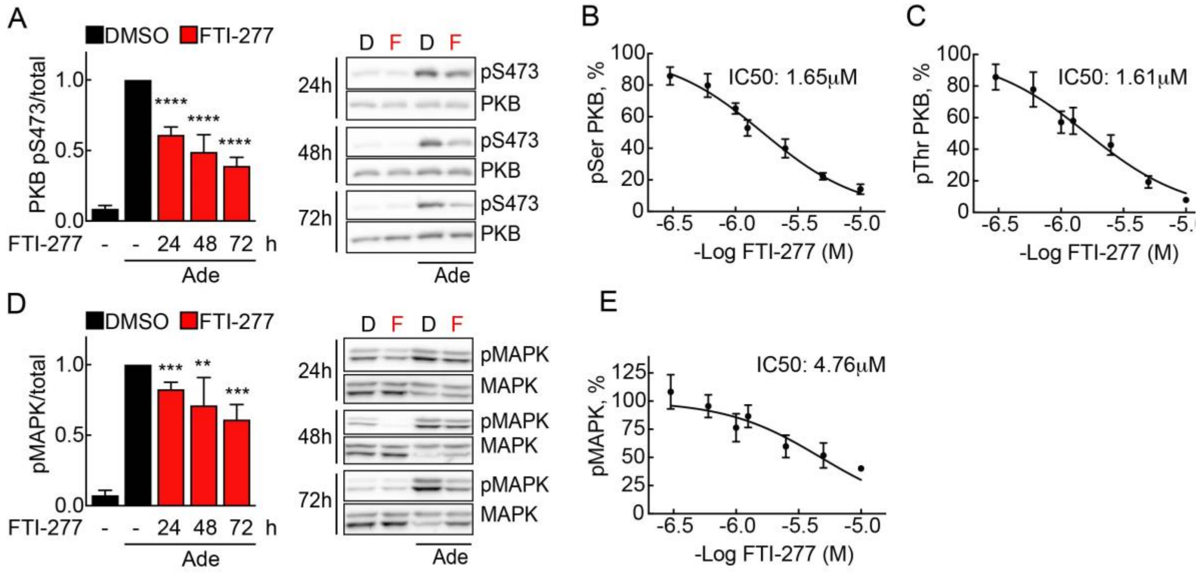

Figure S4 Time- and concentration-dependence of FTI-277 action on mast cells. BMMCs were treated with DMSO or 5µM FTI-277 for 24, 48 or 72 h. Cells were then starved for 4 h in IL3-free medium containing 2% FCS and either DMSO or 5µM FTI-277, followed by stimulation with 2µM adenosine. Phosphorylation of PKB at Ser473 **(A)** and pMAPK **(D)** were determined by Western blotting and normalized to the total PKB or MAPK levels, correspondingly. Each time point contains results from 3-7 experiments. **(B, C, E)** BMMCs were treated with increasing concentration of FTI-277 (0.3µM – 10µM) for 72 h, starved and stimulated as in (A). Levels of phosphorylation of PKB at Ser473 **(B),** pThr308 **(C)** and pMAPK **(E)** were quantified by western blotting, normalized to the total PKB or MAPK levels, and used to determine IC_{50}. Each dilution series was tested in n=5 experiments

and one-way ANOVA with Bonferroni's post hoc test was used to determine statistical significance. **: p≤0.01, ***: p≤0.001; ****: p≤0.0001. D=DMSO; F=FTI-277.

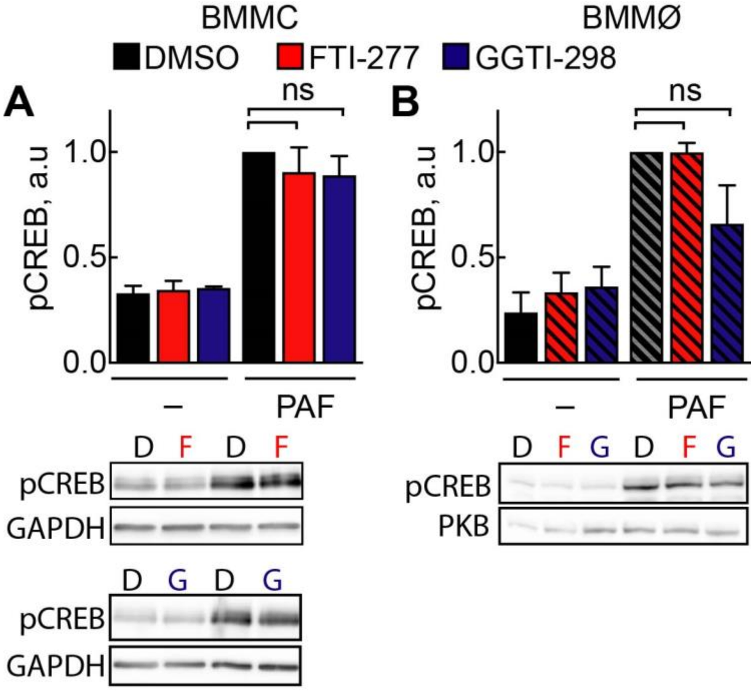

Figure S5. Neither FTI-277 nor GGTI-298 affect PAF-signaling in BMMC or BMMØ. BMMCs and BMMØ were treated with DMSO, 5µM FTI-277 (72 h) or 5µM GGTI (24 h), starved for 4 h and activated with 1µM PAF. Phosphorylation of cyclic AMP-responsive element-binding protein (CREB) at Ser133 was determined by Western blot analysis of cell lysates and normalized GAPDH (BMMC) or PKB (BMMØ). N=3-4 for each data point. Significant relationship was tested with one-way ANOVA with Bonferroni correction. ns: p>0.05. D=DMSO; F=FTI-277; G=GGTI-298.

Table S1. Primer Sequences used for qPCR

Gene	Accession-No	Primer sequence 5’ to 3’
Mm_GAPDH	NM_008084.3	F: CTGCACCACCAACTGCTTAG
		R: CCATCCACAGTCTTCTGGGTG
Mm_p84	AY753194.1	F: ATAGAGCAGGTGGCTAGCGA
		R: CAGAAGGTCACCGACACAGTG
Mm_p101	NM_177320.2	F: GACATCCTACAGGAAGTCCTTCTC
		R: TCAGCGCAATGCCTGTCCAT
Mm_p110g	NM_020272.2	F: CCCTGGTGATCGAGAAATGC
		R: GTCTTGGCGCAGATCATCAC
Mm_NRas	NM_010937.2	F: ATGAGGACAGGCGAAGGGTTC
		R: TCACACTTGTTGCCTACCAGCAC
Mm_KRas4A	XM_006506919.3	F: GATGTGCCTATGGTCCTGGTAGG
		R: GCATCCTCCACTCTCTGTCTTGTC
Mm_KRas4B	NM_021284.6	F: GATGTGCCTATGGTCCTGGTAGG
		R: GCATCGTCAACACCCTGTCTTGTC
Mm_HRas	NM_008284.2	F: GGCAGGGCGTGGAGGATG
		R: GCAGCCAGGACCACTCTCATCG
Mm_RRas	NM_009101.2	F: TGCCATTAACGACAGGCAG
		R: TGTTCCCAACCAACACAATG
Mm_RRas2	NM_025846.2	F: GGCAATAAAGCTGACCTGGA
		R: ATCCTGATCTTTGCCGATG
Mm_MRas	NM_008624.3	F: CCACCAGCTCATTCTGCGTGTCAAGG
		R: CCTTGGTCCCTGGTGACTTTCCTTAGG
Mm_TNFalpha	NM_013693.2	F: ATCCGCGACGTGGAACTG

		R: CGAAGTTCAGTAGACAGAAGA
Mm_IL6	NM_031168.2	F: ACAACCACGGCCTTCCCTACTT
		R: CACGATTTCCCAGAGAACATGTG
Mm_Fnta	NM_008033.3	F: AGCATCGACAGTGGGTCATTC
		R: GACGAAGTGTCTTTGGTTCCAC
Mm_Fntb	NM_145927.2	F: GAGAAGATCCAGGAGGTCTTCAG
		R: CTCATAGGCATCTGTCAGTTGTC
Mm_Pggt1b	NM_172627.3	F: CCATCAAAGAATCCAGGAGCAG
		R: ATCCACACGGCCTAAGTCATCTC

Mm, *mus musculus*; F, forward primer; R, reverse primer

Table S2. Sequences of codon-optimized 3xHA-Ras, used for expression in HEK293 cells

3xHA_H-Ras

```
GGTACCACCATGTATCCTTACGATGTGCCTGACTATGCCTATCCTTACGATGTGC
CAGATTACGCTTATCCCTACGATGTGCCAGATTACGCCAAGCTTGACACAGAGTA
CAAACTGGTGGTGGTGGGAGCTGGCGGAGTCGGGAAGAGCGCACTGACCATCCAG
CTGATTCAGAACCACTTCGTGGACGAGTACGATCCCACAATCGAAGACTCCTATC
GGAAACAGGTCGTGATCGATGGCGAGACATGTCTGCTGGACATTCTGGATACCGC
CGGACAGGAGGAATACAGTGCTATGCGGGATCAGTATATGCGCACAGGGGAAGGC
TTCCTGTGCGTGTTCGCCATTAACAATACTAAGAGTTTTGAGGACATCCATCAGT
ACCGAGAACAGATTAAGAGGGTCAAAGATTCAGACGATGTGCCCATGGTCCTGGT
GGGAAACAAGTGCGACCTGGCCGCTAGAACTGTGGAGAGCCGGCAGGCACAGGAT
CTGGCACGCTCCTACGGGATCCCTTATATTGAAACCTCTGCAAAGACACGACAGG
GCGTCGAGGACGCTTTCTATACCCTGGTGAGGGAAATCAGACAGCACAAGCTGAG
GAAACTGAACCCTCCTGACGAATCTGGCCCTGGCTGTATGTCCTGTAAGTGCGTG
CTGTCTTGACTCGAG
```

3xHA_N-Ras

```
GGTACCACCATGTATCCTTACGATGTGCCTGACTATGCCTATCCTTACGATGTGC
CAGATTACGCTTATCCCTACGATGTGCCAGATTACGCCAAGCTTGACACAGAGTA
CAAACTGGTGGTGGTGGGAGCTGGCGGAGTCGGGAAGAGCGCACTGACCATCCAG
CTGATTCAGAACCACTTCGTGGACGAGTACGATCCCACAATCGAAGACTCCTATC
GGAAACAGGTCGTGATCGATGGCGAGACATGTCTGCTGGACATCCTGGACACCGC
CGGCCAGGAGGAGTACAGCGCCATGCGCGACCAGTACATGCGCACCGGCGAGGGC
TTCCTGTGCGTGTTCGCCATCAACAACAGCAAGAGCTTCGCCGACATCAACCTGT
ACCGCGAGCAGATCAAGCGCGTGAAGGACAGCGACGACGTGCCCATGGTGCTGGT
GGGCAACAAGTGCGACCTGCCCACCCGCACCGTGGACACCAAGCAGGCCCACGAG
CTGGCCAAGAGCTACGGCATCCCCTTCATCGAGACCAGCGCCAAGACCCGCCAGG
GCGTGGAGGACGCCTTCTACACCCTGGTGCGCGAGATCCGCCAGTACCGCATGAA
GAAGCTGAACAGCAGCGACGACGGCACCCAGGGCTGCATGGGCCTGCCCTGCGTG
GTGATGTGACTCGAG
```

3xHA_K-Ras

```
GGTACCACCATGTATCCTTACGATGTGCCTGACTATGCCTATCCTTACGATGTGC
CAGATTACGCTTATCCCTACGATGTGCCAGATTACGCCAAGCTTGACACAGAGTA
CAAACTGGTGGTGGTGGGAGCTGGCGGAGTCGGGAAGAGCGCACTGACCATCCAG
CTGATTCAGAACCACTTCGTGGACGAGTACGATCCCACAATCGAAGACTCCTATC
GGAAACAGGTCGTGATCGATGGCGAGACATGTCTGCTGGACATCCTGGACACCGC
CGGCCAGGAGGAGTACAGCGCCATGCGCGACCAGTACATGCGCACCGGCGAGGGC
TTCCTGTGCGTGTTCGCCATCAACAACACCAAGAGCTTCGAGGACATCCACCACT
ACCGCGAGCAGATCAAGCGCGTGAAGGACAGCGAGGACGTGCCCATGGTGCTGGT
GGGCAACAAGTGCGACCTGCCCAGCCGCACCGTGGACACCAAGCAGGCCCAGGAC
CTGGCCCGCAGCTACGGCATCCCCTTCATCGAGACCAGCGCCAAGACCCGCCAGG
GCGTGGACGACGCCTTCTACACCCTGGTGCGCGAGATCCGCAAGCACAAGGAGAA
GATGAGCAAGGACGGCAAGAAGAAGAAGAAGAAGAGCAAGACCAAGTGCGTGATC
ATGTGACTCGAG
```

Table S3. ORF sequences of GFP-tagged Ras isoforms used for cellular localization assays (C-terminal 25 amino acids in blue)

Start GFP Ras (C-term 25 AA) Restriction site

GFP-HRasCAAX
Plasmid 1672

ATGGTGAGCAAGGGCGAGGAGCTGTTCACCGGGGTGGTGCCCATCCTGGTCGAGC
TGGACGGCGACGTAAACGGCCACAAGTTCAGCGTGTCCGGCGAGGGCGAGGGCGA
TGCCACCTACGGCAAGCTGACCCTGAAGTTCATCTGCACCACCGGCAAGCTGCCC
GTGCCCTGGCCCACCCTCGTGACCACCCTGACCTACGGCGTGCAGTGCTTCAGCC
GCTACCCCGACCACATGAAGCAGCACGACTTCTTCAAGTCCGCCATGCCCGAAGG
CTACGTCCAGGAGCGCACCATCTTCTTCAAGGACGACGGCAACTACAAGACCCGC
GCCGAGGTGAAGTTCGAGGGCGACACCCTGGTGAACCGCATCGAGCTGAAGGGCA
TCGACTTCAAGGAGGACGGCAACATCCTGGGGCACAAGCTGGAGTACAACTACAA
CAGCCACAACGTCTATATCATGGCCGACAAGCAGAAGAACGGCATCAAGGTGAAC
TTCAAGATCCGCCACAACATCGAGGACGGCAGCGTGCAGCTCGCCGACCACTACC
AGCAGAACACCCCCATCGGCGACGGCCCCGTGCTGCTGCCCGACAACCACTACCT
GAGCACCCAGTCCGCCCTGAGCAAAGACCCCAACGAGAAGCGCGATCACATGGTC
CTGCTGGAGTTCGTGACCGCCGCCGGGATCACTCTCGGCATGGACGAGCTGTACA
AGTCCGGACTCAGATCT(BglII)CAGCACAAGCTGAGGAAACTGAACCCTCCTG
ACGAATCTGGCCCTGGCTGTATGTCCTGTAAGTGCGTGCTGTCTTGAGAATTC(E
coRI)

GFP-NRasCAAX
Plasmid 1673

ATGGTGAGCAAGGGCGAGGAGCTGTTCACCGGGGTGGTGCCCATCCTGGTCGAGC
TGGACGGCGACGTAAACGGCCACAAGTTCAGCGTGTCCGGCGAGGGCGAGGGCGA
TGCCACCTACGGCAAGCTGACCCTGAAGTTCATCTGCACCACCGGCAAGCTGCCC
GTGCCCTGGCCCACCCTCGTGACCACCCTGACCTACGGCGTGCAGTGCTTCAGCC
GCTACCCCGACCACATGAAGCAGCACGACTTCTTCAAGTCCGCCATGCCCGAAGG
CTACGTCCAGGAGCGCACCATCTTCTTCAAGGACGACGGCAACTACAAGACCCGC
GCCGAGGTGAAGTTCGAGGGCGACACCCTGGTGAACCGCATCGAGCTGAAGGGCA
TCGACTTCAAGGAGGACGGCAACATCCTGGGGCACAAGCTGGAGTACAACTACAA
CAGCCACAACGTCTATATCATGGCCGACAAGCAGAAGAACGGCATCAAGGTGAAC
TTCAAGATCCGCCACAACATCGAGGACGGCAGCGTGCAGCTCGCCGACCACTACC
AGCAGAACACCCCCATCGGCGACGGCCCCGTGCTGCTGCCCGACAACCACTACCT
GAGCACCCAGTCCGCCCTGAGCAAAGACCCCAACGAGAAGCGCGATCACATGGTC
CTGCTGGAGTTCGTGACCGCCGCCGGGATCACTCTCGGCATGGACGAGCTGTACA
AGTCCGGACTCAGATCT(BglII)CAGTACCGCATGAAGAAGCTGAACAGCAGCG
ACGACGGCACCCAGGGCTGCATGGGCCTGCCCTGCGTGGTGATGTGAGAATTC(E
coRI)

GFP-KRasCAAX
Plasmid 1674

ATGGTGAGCAAGGGCGAGGAGCTGTTCACCGGGGTGGTGCCCATCCTGGTCGAGC
TGGACGGCGACGTAAACGGCCACAAGTTCAGCGTGTCCGGCGAGGGCGAGGGCGA
TGCCACCTACGGCAAGCTGACCCTGAAGTTCATCTGCACCACCGGCAAGCTGCCC
GTGCCCTGGCCCACCCTCGTGACCACCCTGACCTACGGCGTGCAGTGCTTCAGCC
GCTACCCCGACCACATGAAGCAGCACGACTTCTTCAAGTCCGCCATGCCCGAAGG
CTACGTCCAGGAGCGCACCATCTTCTTCAAGGACGACGGCAACTACAAGACCCGC
GCCGAGGTGAAGTTCGAGGGCGACACCCTGGTGAACCGCATCGAGCTGAAGGGCA
TCGACTTCAAGGAGGACGGCAACATCCTGGGGCACAAGCTGGAGTACAACTACAA
CAGCCACAACGTCTATATCATGGCCGACAAGCAGAAGAACGGCATCAAGGTGAAC
TTCAAGATCCGCCACAACATCGAGGACGGCAGCGTGCAGCTCGCCGACCACTACC
AGCAGAACACCCCCATCGGCGACGGCCCCGTGCTGCTGCCCGACAACCACTACCT
GAGCACCCAGTCCGCCCTGAGCAAAGACCCCAACGAGAAGCGCGATCACATGGTC
CTGCTGGAGTTCGTGACCGCCGCCGGGATCACTCTCGGCATGGACGAGCTGTACA
AGTCCGGACTCAGATCT(BglII)CGCAAGCACAAGGAGAAGATGAGCAAGGACG
GCAAGAAGAAGAAGAAGAAGAGCAAGACCAAGTGCGTGATCATGTGAGAATTC(E
coRI)

Table S4. Antibodies used for immunoblotting

**Antibody**		**Company/Cat. Nr.**
p110g (aa 97-335)	Clone H1 (*the russian*), mouse IgG2a	Alexis, 804-230-L001 (currently no commercial source)
p101 (aa 508)	clone D32A5, rabbit IgG	Cell Signaling, 5569S
p84 (aa 1-162)	Polyclonal, rabbit serum	Wymann Lab, rabbit 1, serum1
K-Ras (aa 54-189)	Clone F234, mouse IgG2a	Santa Cruz, Sc-30
N-Ras	Clone F155, mouse IgG1	Santa Cruz, sc31n
H-Ras	Clone Y132, rabbit IgG	Abcam, ab32417
R-Ras (aa 11-31)	Rabbit IgG	Abcam, ab47536
Lamin	Polyclonal, Goat IgG	Santa Cruz, sc-6215
Pre-Lamin	Polyclonal, goat IgG	Santa Cruz, sc-6214
Rap1A/B	Clone 26B4, rabbit IgG	Cell signaling, 2399
pS473 PKB	clone 193H12, Rabbit IgG	Cell Signaling, 4058
pT308 PKB	clone 244F9, Rabbit IgG	Cell Signaling, 4056
PKB	clone 40D4, Mouse IgG1	Cell Signaling, 2920
pMAPK (pERK-1&2)	mouse monoclonal anti-MAPK, acrivated, diphosphorylated ERK-1&2, clone MAPK-YT, ascites fluid IgG1	Sigma-Aldrich, M8159
MAPK (ErK-1, aa351-368)	polyclonal rabbit anti-ERK-1 (aa351-368)	Sigma-Aldrich, M7927
pS133 CREB	Clone 87G3, rabbit IgG	Sigma-Aldrich, 9198
GAPDH	clone GAPDH-71.1, mouse IgM	Sigma-Aldrich, G8795
Tubulin	clone DM1A, mouse IgG1	Sigma-Aldrich, T9026
Anti-Rabbit IgG-Peroxidase conjugate	Polyclonal, Goat	Sigma-Aldrich, A6154
Anti-Rabbit IgG Peroxidase conjugate	Polyclonal, Goat	Sigma-Aldrich, A4416

Anti-Goat IgG-HRP conjugate	Polyclonal, Donkey	Santa Cruz, sc-2020
